# Controllable Synthesis of Hybrid Dendrimers Composed of a Carbosilane Core and an Aromatic Shell: Does Size Matter?

**DOI:** 10.3390/ijms232415461

**Published:** 2022-12-07

**Authors:** Sofia N. Ardabevskaia, Elena S. Chamkina, Irina Yu. Krasnova, Sergey A. Milenin, Ekaterina A. Sukhova, Konstantin L. Boldyrev, Artem V. Bakirov, Olga A. Serenko, Zinaida B. Shifrina, Aziz M. Muzafarov

**Affiliations:** 1 N.S. Enikolopov Institute of Synthetic Polymeric Materials, Russian Academy of Sciences, 70 Profsouznaya St., 117393 Moscow, Russia; 2 Research Laboratory of New Silicone Materials and Technologies, Tula State Lev Tolstoy Pedagogical University, 125 Lenin Ave., Building 4, 300026 Tula, Russia; 3 A.N. Nesmeyanov Institute of Organoelement Compounds, Russian Academy of Sciences, 28 Vavilov St., 119991 Moscow, Russia; 4 National Research Center “Kurchatov Institute”, Akademika Kurchatova pl., 1, 123182 Moscow, Russia

**Keywords:** hybrid dendrimers, “click” reactions, ordered structures, core/shell macromolecules

## Abstract

The controllable synthesis of novel hybrid dendrimers composed of flexible and rigid components was accomplished via effective Cu-catalyzed azide–alkyne cycloaddition (“click”) reaction between azide-functionalized carbosilane cores of two generations and monoethynyl-substituted hexaphenylbenzene dendron. A comprehensive analysis of the thermal and phase behavior of dendrimers allows us to detect a similar performance of dendrimers of both generations which, in our opinion, can be due to the similar ratio of rigid and flexible blocks in the dendrimers regardless the generation of carbosilane cores. The propensity to crystallization and ordering after the annealing procedure was confirmed by DSC and SWAXS. We found that hybrid dendrimers have a tendency to order depending on their constituents of different structures. This is in contrast to homogeneous dendrimers whose propensity to order is determined by the dendrimer molecule as a whole.

## 1. Introduction

Hybrid macromolecules are a class of modern materials that combine the advantages of their constituent components and manifest the synergistic effect. The proper combination of different blocks in the complex macromolecular structure allows for creation polymer systems with desired characteristics useful for further applications. The behavior of such systems, including self-assembly, morphology, and physical properties is determined by architecture, rigidity, size, etc., of the building segments. These structures include core/shell nanoparticles and nanostructures [1,2,3], different block molecules [4,5], rod-coil copolymers [6,7] and other systems [8,9,10]. Among these hybrid structures, dendritic systems occupy a niche that is mostly limited by flexible constituents [11,12,13,14]. Recently, we synthesized a group of core/shell dendrimers with controllable rigidity by a combination of rigid polyphenylene dendritic segments and flexible carbosilane hyperbranched blocks linked in different sequences. Hybrid dendrimers consisting of a flexible carbosilane core and a rigid polyphenylene shell were tailored through the Diels–Alder reaction [15]. Vice versa dendrimers composed of a rigid polyphenylene core and a flexible carbosilane shell were synthesized through the Cu-catalyzed azide–alkyne cycloaddition “click” reaction between the core and shell [16]. The strong effect of the chemical structure of the shell, block size, and rigidity on thermophysical characteristics, molecular behavior, and ordering ability for the final hybrid dendrimers was established. While the dendrimers with a carbosilane core and polyphenylene shell do not tend to order, the dendrimers with a flexible carbosilane shell demonstrate an ordering of mesophase dendrimer structure that decreases with the increasing carbosilane periphery size (generation). The developed approach to complex rigid-flexible dendrimers opens up the prospect for a controllable synthesis of hybrid dendrimers with a desired structure and finely-tuned properties.

Here, we report a further development of the controlled synthesis of the dendrimers constructed from the first- or second-generation flexible carbosilane cores and decorated with rigid hexaphenylbenzene (HPB) dendrons linked to the core through the rigid triazole groups. Unlike the similar hybrid dendrimers synthesized by us earlier via the Diels–Alder approach [15], the introduction of rigid HPB units in the dendrimer shell through the “click” reaction between azide-terminated carbosilane dendrimers and monoethynyl-terminated HPB promoted the ordering of the hybrid system. The effect of the number and density of HPB groups in the dendrimer shell on the degree of crystallinity of macromolecules and the packing density in the crystal structure, as well as phase and thermal behavior, were demonstrated.

## 2. Results and Discussion

### 2.1. Synthesis of Hybrid Dendrimers

**Scheme 1:**
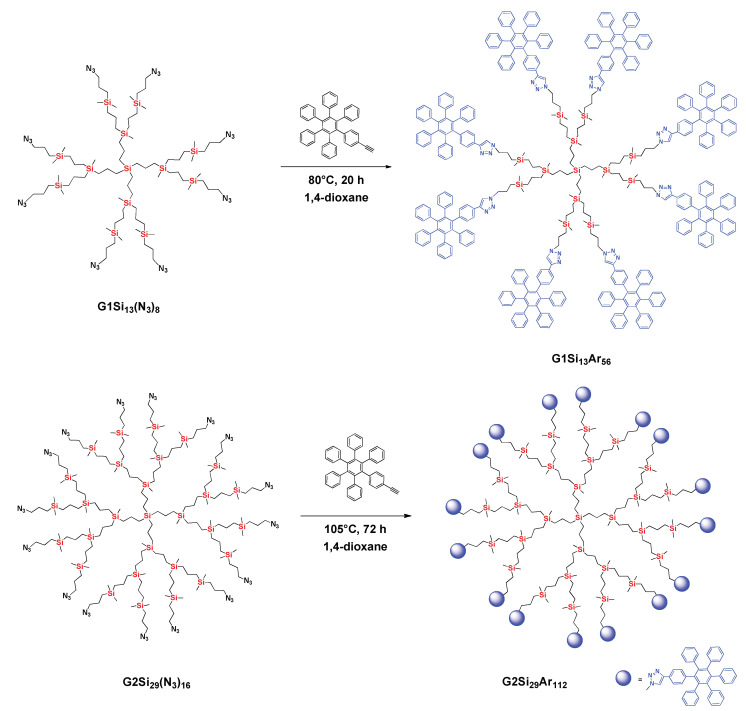
Synthesis of the hybrid dendrimers **G1Si_13_Ar_56_** and **G2Si_29_Ar_112_**.

Carbosilane dendrimers with HPB peripheral units were obtained via the CuAAC “click” reaction between the azide-terminated dendrimers **G1Si_13_(N_3_)_8_** or **G2Si_29_(N_3_)_16_** and monoethynyl-containing HPB dendrons (Scheme 1). The synthesis of azide-terminated dendrimers was accomplished through the modified procedures developed by us earlier via a two-step approach. First, carbosilane dendrimers were hydrosilylated with 3-chloropropyldimethylsilane followed by a replacement of the halogen for azide groups by sodium azide (Schemes S1 and S2, Figures S1–S7, ESI). The complete substitution was for 24 h and 50 h for the first and second generation, respectively [17,18,19]. Synthesis of ethynyl-terminated HPB (Scheme S3, ESI) was carried out according to the procedures presented in [20]. The structures of final hybrid dendrimers were confirmed by ^1^H, ^13^C, ^29^Si NMR spectroscopy (Figures S8–S13, ESI), MALDI-ToF MS and elemental analysis. The ^1^H NMR spectrum of the as-synthesized dendrimer **G1Si_13_Ar_56_** (Figure S8, ESI) showed the absence of the signal at 3.24 ppm corresponding to the methylene protons near azide group (–CH_2_–N_3_) of the parent dendrimer, and the appearance of the signal at 7.50 ppm corresponding to the triazole proton and multiplet at 4.15–4.31 ppm for methylene protons near the triazole group. This means that all azide groups were converted into triazole groups by the azide–alkyne “click” reaction with ethynyl-terminated HPB dendrons and an ideal dendrimer molecule was formed (Scheme 1). To achieve the highest possible transformation degree of azide groups into triazole groups for the second-generation dendrimer **G2Si_29_Ar_112_**, the reaction temperature was increased to 105 °C, and the time of the reaction was extended to 72 h. This allowed us to convert up to 95% of the azide groups of the parent second-generation dendrimer into triazole groups, according to NMR data (Figure S11, ESI). According to GPC analysis, the purity of as-synthesized dendrimers was 99% and 95%, for the first and the second generation, respectively. (Figure S14, ESI). A mixture of matrix (2,5-dihydroxybenzoic acid) and sodium chloride as an ionizing dopant was used for the MALDI-ToF measurements. MALDI-ToF mass spectrum of the dendrimer **G1Si_13_Ar_56_** showed the molecular ion (6337 Da), consistent with the theoretically calculated mass of dendrimer (6313 Da) plus Na^+^ (Figure S15, ESI). For dendrimer **G2Si_29_Ar_112,_** the signal of the target dendrimer was detected by the MALDI-ToF method with α-cyano-4-hydroxycinnamic acid as a matrix and LiCl as a dopant. The spectrum showed molecular ion (12940 Da), consistent with the calculated dendrimer mass (12939 Da) (Figure S16, ESI). In the following discussion, the **G1Si_13_Ar_56_** and **G2Si_29_Ar_112_** are denoted as G1 and G2, respectively.

### 2.2. Thermal Properties of Hybrid Dendrimers

**Figure 1:**
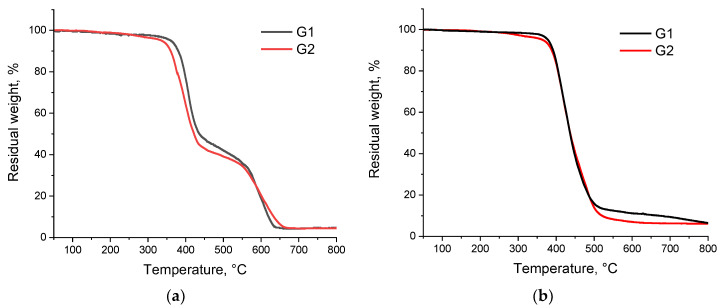
TGA traces of dendrimers G1 and G2 in air (**a**) and in argon (**b**) at a heating rate of 10°/min.

The thermal stability of hybrid dendrimers G1 and G2 was determined by thermogravimetric analysis (TGA) in air and in argon. Earlier [15], we found that the aromatic shell mainly determined the character of the thermal decomposition process in hybrid dendrimers composed of a flexible interior and rigid shell. For example, for the dendrimer in [15] composed of one generation of flexible carbosilane cores and two generations of rigid phenylene terminal blocks, the onset of the decomposition temperature was 100 °C higher than for the dendrimer composed of the same carbosilane core and one generation of the rigid polyphenylene shell. That is, the larger (thicker) rigid shell and the high decomposition temperature of the hybrid dendrimer. For dendrimers reported here, the weight-loss curves of both G1 and G2 dendrimers in air had two modes (Figure 1a). The first mode started at around 360 °C (5% weight loss) for G1 and 340 °C (5% weight loss) for G2 and was responsible for the decomposition of the carbosilane structure. At around 450 °C, the plateau was observed followed by the second stage onsets at 550 °C. The second stage of thermal decomposition was responsible for the decomposition of the aromatic outer layer of the dendrimers [21,22]. The process finished at 650 °C with a residual coke of 5%. The thermal stability of dendrimers in argon was slightly higher than in air, and the decomposition proceeded in one step starting from the 380–390 °C and finished at 600 °C with a residual coke of below 10% for both dendrimers. As in the case of TGA in air, the onset of the decomposition temperature for both dendrimers at 380 °C corresponded to thermal destruction of carbosilane bonds (Figure 1b). As one can see from TGA traces, the thermal decomposition of G1 and G2 proceeded in analogous manner. Thus, unlike abovementioned dendrimers synthesized by us in previous work [15], for G1 and G2 dendrimers in this study, there were no crucial differences in thermal stability. This is true for measurements in both air and in argon. Although the amount of the rigid terminal blocks increased proportionally with an increase in the generation of carbosilane cores, the ratio between of rigid and flexible components of the system remained constant for G1 and G2. We believe this is a key factor for the absence of the significant differences in the thermal decomposition behavior between dendrimers. At the same time, the differences in the TGA profiles in argon and air showed the contribution from oxidation in the decomposition process.

### 2.3. The Phase Behavior of the Dendrimers

**Figure 2:**
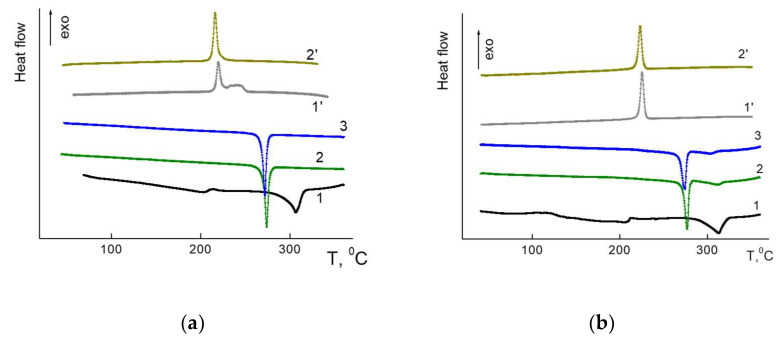
DSC traces for the first, second and third heating–cooling cycles for G1 (**a**) and G2 (**b**).

According to DSC “click”, hybrid dendrimers with terminal HPB units showed a melting and a crystallization peak during heating–cooling cycles (Figure 2). This contrasts with previously-synthesized hybrid dendrimers with polyphenylene shells linked to a carbosilane core via the hexadiene cycle, demonstrating glass-transition temperatures [15]. The thermal behavior of G1 and G2 dendrimers was found to be similar regardless of the generation number. In the first heating to 350 °C both of the as-received dendrimers showed wide peaks corresponding to the cold crystallization and melting with extremum at 213 °C and 306 °C, and at 212 °C and 312 °C, for G1 and G2, respectively. Further cooling of G1 showed two crystallization peaks. The narrow peak at 220 °C is accompanied by a diffuse peak at 240 °C. Here, it should be noted that the ΔH of crystallization and melting were close (Table 1). For the G2 at cooling, a narrow crystallization peak was observed at 225 °C only. For this dendrimer, the fusion heat for crystallization was somewhat lower than for melting (Table 1).

At the second heating–cooling cycle, the behavior of both dendrimers changed. While the peaks of cold crystallization were absent, the new obtained phases melted at lower temperatures (273–276 °C) than in the first cycles. Additionally, for G2, the residue effect of low intensity was observed at 311 °C, close to the first cycle melting. At cooling, the temperature peaks of crystallization mostly coincided with those of first cycle for both samples (Table 1, Figure 2). The ΔH of melting and crystallization for the first and second cycles also coincided for both dendrimers. However, it is worth noting that generally, in the second cycle, the ΔH was higher for G1 compared with G2, indicating a higher content of ordered phase in G1. In the third heating cycle, the phase behavior of both dendrimers coincided with that in the second cycle.

**Figure 3:**
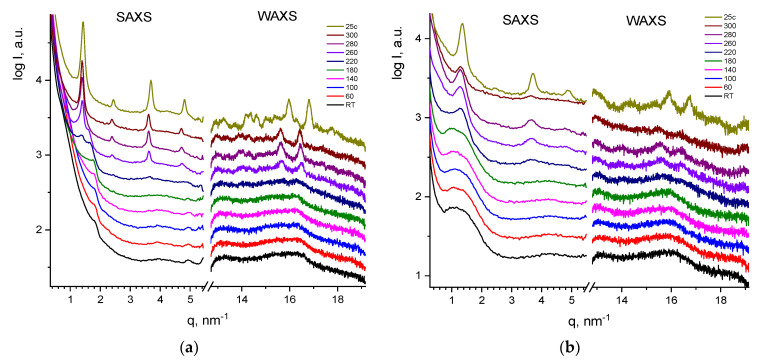
SWAXS curves for as-received G1 (**a**) and G2 (**b**) at varied temperatures during first and second heating runs (up 300 °C) with subsequent cooling. The curves are shifted vertically for clarity.

The change in temperature and the narrowing of the melting peak of dendrimers in the subsequent heating–cooling cycles allows us to suggest that the as-received samples of dendrimers show a nonequilibrium polycrystalline structure. The gradual heating of samples to 350 °C was accompanied by annealing and recrystallization of metastable ensembles of dendrimers. This assumption was supported by the SAXS and WAXS data (Figure 3).

The as-received G1 and G2 showed only a small-angle reflection (SAXS) and a halo in wide angles, indicating weak ordering (Figure 3a,b). However, heating above 220 °C led to cold crystallization and resulted in a set of small- and wide-angle reflections, indicating the appearance of a powder crystalline pattern. Further, up to 300 °C, no structural changes in G1 were observed. However, for G2, one can observe the drop of the reflection intensity, indicating the onset of an isotropic state (Figure 3b).

**Figure 4:**
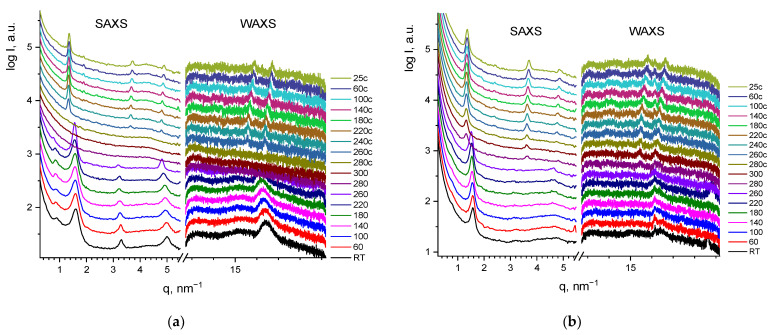
SWAXS curves for annealed G1 (**a**) and G2 (**b**) at varied temperatures. The curves are shifted vertically for clarity.

The samples that underwent an annealing procedure (heating up to 350 °C) also showed a stable crystalline structure (Figure 4a,b). For G1, an ordered liquid crystal phase with a set of small-angle reflections d_1_^2^:d_2_^2^:d_3_^2^:d_4_^2^ = 1:3:13:30 was observed. The interplanar spacing was 4 ± 0.05 nm. The heating of the annealed sample up to 300 °C led to the melting of a crystalline phase, while the subsequent cooling resulted in the phase recovery. The analogous scenario was observed for G2 dendrimer (Figure 4b). Thus, with these results we confirmed the reversible behavior of dendrimers previously detected by DSC.

While the phase behavior was similar for both dendrimers regardless of the generation number, the lower molecular weight sample yielded narrower reflections, possibly forming larger crystallites of ~27 nm G1 versus 11 nm for G2 as assessed from the Scherrer equation. While the SWAXS experiments confirmed the crystal nature of the obtained annealed phase, it was not possible to index the obtained peak set and determine the crystal system due to the gap in the experimental data between the small- and wide-angle detectors.

**Figure 5:**
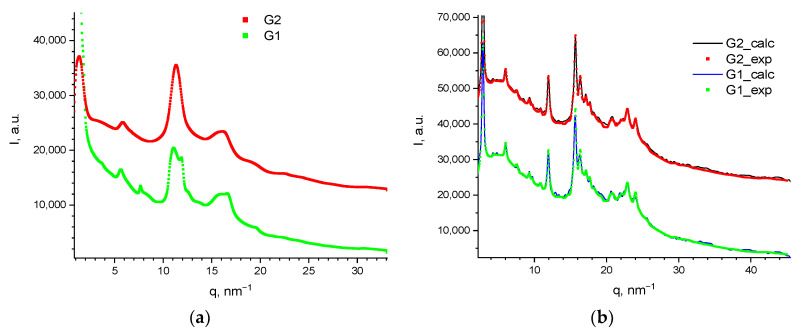
WAXS curves for G1 and G2, as-received (**a**), and annealed samples at 300 °C (**b**). Solid lines show the Pawley refinement, and dots are for the experimental data. Curves are shifted vertically for clarity.

Therefore, we studied the as-received and annealed samples of G1 and G2 dendrimers at BIOSAXS beamline at Kurchatov synchrotron in the transmission WAXS powder regime (Figure 5a,b). Obtained curves confirmed our conclusions on the occurring crystallization.

**Figure 6:**
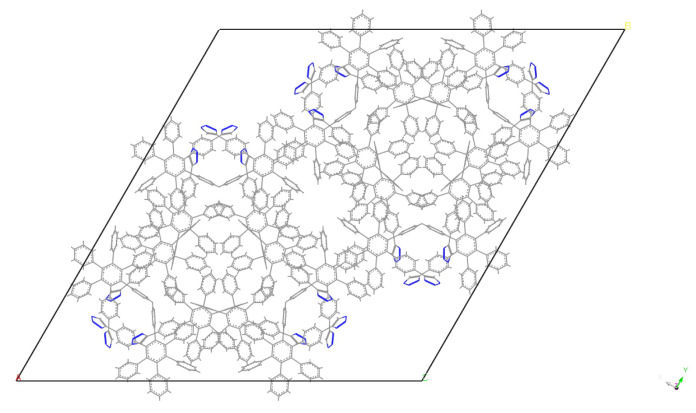
Molecular packing of aromatic fragments to the crystal cell. Only the HPB fragments are shown in the presented packing solutions.

Interestingly, no considerable differences in the diffraction curves between the dendrimers of various generations was noted. Thus, it can be concluded that only the crystallization of the aromatic terminal groups occurred, while the flexible core did not participate in this process. The diffraction pattern of ~30 peaks was indexed as a hexagonal lattice of P6cc symmetry with a = b = 50 Å, and c = 11.6 Å with R_wp_ = 1.4%. The parameters of the cell did not change substantially with the varying of the core. While the diameters of G1 and G2 dendrimer molecules were roughly 62 and 68 Å, respectively, and the stacking length of such “propeller”-shaped functional aromatic groups is 9–10 Å as reported elsewhere [23,24,25], the flat organization of the molecules in the crystal cell can be assumed (Figure 6). The degree of crystallinity was roughly estimated at 50–57%. This value of crystallinity can be considered as a maximum of the crystallinity of the dendrimers synthesized. As can be seen from the DSC data (Table 1), the crystallinity for G2 was lower than that for G1. The calculation of the specific fusion heat with respect to the crystallizing shell content produced 46 J/g and 32 J/g, for G1 and G2, respectively. These results seem relevant because in the case of G1, the mass ratio of the crystallizing shell to the amorphous core was slightly larger than in case of G2. We assume that as the generation number of the flexible core increases, the crystallinity degree of the hybrid dendrimer will slightly decrease since the content of the amorphous part of molecules gradually increases relatively to crystallizing part.

To solve the crystal cell, we utilized the Reflex module of the Biovia Material Studio software package [26]. After placing the G1 and G2 dendrimers in the obtained crystal cell, a simulated annealing run loop was conducted several times to meet both the minimal energy due to close-contact penalty and the R_wp_ value of discrepancy between the simulated and the experimental diffraction curves. Since the branched macromolecules such as dendrimers possess a high number of torsion freedom degrees, only HPB fragments were taken into consideration, since no remarkable difference can be seen comparing the dendrimers of various generations. Such an approach of omitting the non-aromatic fragments was already applied for similar objects [27]. To begin the solving process, the initial packing of similar aromatic groups was accounted [28]. The obtained packing possesses a high symmetry as required by the space group P6CC determined on one hand and provides the strong π-π stacking energy of the aromatic groups on the other. This interaction comes not only from the neighboring branches, but also from the neighboring dendrimers, and leads to the crystalline columnar organization that appears after annealing.

In previous work [15] we have shown that the inverse dendrimers with a rigid polyphenylene core and a soft shell can only form mesophases and are not able to crystallize. The current study shows the ability of HPB to crystallize regardless of the dendrimer generation number. However, removing even one benzene ring from the HPB terminal groups results in the inability to crystallize (data not shown). One possible reason for this is the high energy rotational barrier of the aromatic rings, attached to the central one, which defines the special orientation of such groups. Removing of one of these end fragments decreases this barrier, increases the molecular torsion mobility, and lowers the ability of the material to crystallize.

## 3. Materials and Methods

### 3.1. Materials

Allyl chloride, 98% (CAS 107-05-1), tetra-n-butylammonium fluoride (1 M solution in THF, CAS 429-41-4), copper (I) iodide, 98% (CAS 7681-65-4) and *N*,*N*-Dimethylformamide, 99.9% (CAS 68-12-2) were obtained from ABCR. Sodium azide (CAS 26628-22-8), diphenylether, 99% (CAS 101-84-8) and triethylamine, 99% (CAS 121-44-8) were obtained from Acros Organics. Karstedt’s catalyst (CAS 11057-89-9), diphenylacetylene, 98% (CAS 501-65-5), chlorodimethylsilane, 98% (CAS 1066-35-9) and lithium aluminum hydride (CAS 16853-85-3) were obtained from Sigma-Aldrich. All chemicals were used as received.

The solvents were purified by distillation under reduced pressure in argon.

### 3.2. Synthetic Procedures

All reactions were carried out in inert atmosphere. The allyl-terminated dendrimer was synthesized according to procedures developed by us earlier [29,30]. 3-Chloropropyldimethylsilane was obtained according to the previously-described method [31].

#### 3.2.1. Synthesis of the Monoethynyl HPB Dendron

The HPB dendron was synthesized according to the procedure presented in [20] (Scheme S3, ESI).

#### 3.2.2. Synthesis of the Azide-Terminated Dendrimers

##### Synthesis of ***G1Si_13_(Cl)_8_***

The allyl-terminated **carbosilane** dendrimer G1Si_5_All_8_ (1 g, 1.4 × 10^−3^ mol) was dissolved in 5 mL of dry toluene, then (3-chloropropyl)dimethylsilane (1.7 g, 1.2 × 10^−2^ mol) and Karstedt’s catalyst were added to the solution. The obtained mixture was stirred at room temperature for 48 h. The reaction was monitored by ^1^H NMR. The reaction mixture was concentrated under reduced pressure (80 °C/0.5 mbar). The product was obtained as a colorless oil with a 99% yield (2.54 g, 99% of purity according to GPC). ^1^H NMR (300 MHz, CDCl_3_): δ 3.49 (m, 16H, CH_2_-Cl), 1.83–1.70 (m, 16H, CH_2_-CH_2_-Cl), 1.32 (m, 24H, Si-CH_2_-CH_2_-CH_2_-Si), 0.52–0.62 (m, 64H, Si-CH_2_), −0.07 - 0.08) (m, 60H, Si-CH_3_).

##### Synthesis of ***G2Si_29_(Cl)_16_***

**G2Si_29_(Cl)_16_** was obtained similarly to the dendrimer **G1Si_13_(Cl)_8_** from G2Si_13_All_16_. The product was obtained as a colorless oil with a 99% yield (3.35 g, 99% of purity according to GPC). ^1^H NMR (300 MHz, CDCl_3_): δ 3.49 (m, 32H, CH_2_-Cl), 1.79–1.72 (m, 32H, CH_2_-CH_2_-Cl), 1.32 (m, 56H, Si-CH_2_-CH_2_-CH_2_-Si), 0.52–0.62 (m, 144H, Si-CH_2_), 0.006–0.07 (m, 132H, Si-CH_3_).

##### Synthesis of ***G1Si_13_(N_3_)_8_*** [18]

The chlorine-terminated dendrimer G1Si_13_(Cl)_8_ (2.54 g, 1.4 × 10^−3^ mol) was dissolved in 8 mL of dry DMF, then NaN_3_ (0.96 g, 1.4 × 10^−2^ mol) was added to the solution. The obtained mixture was stirred at 80 °C for 24 h. The reaction was monitored by ^1^H NMR. The reaction mixture was filtered through silica gel with hexane and concentrated under reduced pressure (80 °C/0.5 mbar). The product was obtained as a colorless oil with a 95% yield (2.47 g, 99% of purity according to GPC). ^1^H NMR (300 MHz, CDCl_3_): δ 3.22 (m, 16H, CH2-N_3_), 1.63–1.52 (m, 16H, C*H_2_*-CH_2_-Cl), 1.30 (m, 24H, Si-CH_2_-C*H_2_*-CH_2_-Si), 0.62–0.49 (m, 64H, Si-CH_2_), −0.02, −0.07 (m, 60H, Si-CH_3_). ^13^C NMR (77.5 MHz, CDCl_3_): δ 77.54, 77.11, 76.69, 54.63, 54.37, 23.78, 23.15, 20.03, 20.00, 19.26, 18.89, 18.71, 18.52, 17.80, 15.48, 12.64, 1.33, 0.35, 0.20, −3.28, −3.32, −3.61, −4.71, −4.88. ^29^Si NMR (59.6 MHz, CDCl_3_): δ 7.81, 2.26, 1.16, 0.73, −7.37.

##### Synthesis of ***G2Si_29_(N_3_)_16_***

**G2Si_29_(N_3_)_16_** was obtained similarly to the dendrimer **G1Si_13_(N_3_)_8_**. The chlorine-terminated dendrimer G2Si_29_(Cl)_16_ (3.35 g, 8.6 × 10^−4^ mol) was dissolved in 8 mL of dry DMF, then NaN_3_ (1.15 g, 1.8 × 10^−2^ mol) was added to the solution. The obtained mixture was stirred at 80 °C for 50 h. The target dendrimer was obtained as a colorless oil with an 87% yield (2.96 g, 95% of purity according to GPC). ^1^H NMR (300 MHz, CDCl_3_): δ 3.22 (m, 32H, CH_2_-N_3_), 1.63–1.52 (m, 32H, CH_2_-CH_2_-N_3_), 1.29 (m, 56H, Si-CH_2_-CH_2_-CH_2_-Si), 0.55 (m, 144H, Si-CH_2_), −0.03 - 0.08 (m, 132H, Si-CH_3_). ^13^C NMR (77.5 MHz, CDCl_3_): δ 54.42, 31.51, 23.59, 22.58, 19.84, 18.68, 18.35, 14.08, 12.42, −3.45, −5.01. ^29^Si NMR (59.6 MHz, CDCl_3_): δ 2.08, 0.98, −7.51.

#### 3.2.3. General Procedure for the “Click” Reactions

A mixture of the appropriate azide-terminated dendrimer and ethynyl-terminated HPB dendron in dry dioxane with TEA was stirred until the dissolution of all compounds. Then, copper (I) iodide was added. The resulting suspension was heated for a given time. The cooled reaction mixture was then concentrated. The residue was dissolved in CH_2_Cl_2_ and washed with a saturated aqueous solution of NH_4_Cl until the water layer became colorless. Then, the organic layer was washed with water until neutral, dried with anhydrous Na_2_SO_4_ overnight, and concentrated under reduced pressure. The excess of dendron was separated using column chromarography on silica gel with CH_2_Cl_2_ as the eluent Then, the eluent was changed to a CH_2_Cl_2_: EtOH (10:1) mixture to obtain the target dendrimer.

##### Synthesis of ***G1Si_13_Ar_56_***

The synthesis was performed according to the general procedure using **G1Si_13_(N_3_)_8_** (0.080 g, 0.043 mmol), the ethynyl-terminated HPB dendron (0.253 g, 0.453 mmol), CuI (0.007 g, 0.037 mmol), 1,4-dioxane (6 mL), and TEA (1.5 mL). The reaction mixture was stirred at 80 °C for 20 h. The product was obtained as a white powder with a 73% yield (0.200 g, 0.032 mmol, 99% of purity according to GPC). ^1^H NMR (CDCl_3_, 400.13 MHz): δ −0.11–0.01 (m, 15H, CH_3_–Si); 0.42–0.64 (m, 16H, –CH_2_–Si); 1.24–1.35 (m, 6H, –CH_2_–); 1.76–1.90 (m, 4H, –CH_2_–CH_2_–N); 4.15–4.31 (m, 4H, –CH_2_–N); 6.72–7.07 (m, 54H, Ar); 7.33 (d, 4H, Ar); 7.50 (s, 2H, =CH–N). ^13^C NMR (CDCl_3_, 100 MHz): −4.90 (s); −3.33 (s); 0.26 (s); 12.48 (s); 15.10 (s); 17.67 (s); 18.40 (s); 18.74 (s); 19.15 (s); 19.81 (s); 24.68 (s); 25.36 (s); 29.66 (s); 53.80 (s, NCH_2_); 120.17 (s, NCH); 124.15 (s); 125.20 (s); 126.56 (s); 126.70 (s); 131.34 (s); 131.92 (s); 139.62 (s); 140.24 (s); 140.44 (s); 141.13 (s); 147.02 (s, NCCH). ^29^Si NMR (CDCl_3_, 59 MHz): −7.54 (Si); 0.97 (Si–CH_3_); 2.11 (Si(CH_3_)_2_).

Elemental analysis. Calcd. (%) for C_432_H_420_N_24_Si_13_: C 82.19, H 6.71, N 5.32, Si 5.78; found: C 81.88, H 6.87, N 5.21, Si 5.63.

##### Synthesis of ***G2Si_29_Ar_112_***

The synthesis was performed according to the general procedure using **G2Si_29_(N_3_)_16_** (0.060 g, 0.015 mmol), the ethynyl-terminated HPB dendron (0.250 g, 0.447 mmol), CuI (0.007 g, 0.037 mmol), 1,4-dioxane (5 mL), and TEA (0.8 mL). The reaction mixture was stirred at 105 °C for 72 h. The product was obtained as a white powder with a 70% yield (0.136 g, 0.011 mmol, 95% of purity according to GPC). ^1^H NMR (CDCl_3_, 300 MHz): −0.14–0.07 (m, 33H, CH_3_–Si); 0.38–0.74 (m, 36H, –CH_2_–Si); 1.18–1.41 (m, 14H, –CH_2_–); 1.71–1.93 (m, 8H, –CH_2_–CH_2_–N); 4.07–4.35 (m, 8H, –CH_2_–N); 6.68–7.04 (m, 108H, Ar); 7.31 (d, 8H, Ar); 7.40 (s, 4H, =CH–N). ^13^C NMR (CDCl_3_, 75 MHz): −5.03 (s); −4.94 (s); −3.45 (s); 12.35 (s); 18.34 (s); 18.46 (s); 18.67 (s); 18.96 (s); 19.75 (s); 25.28 (s); 53.19 (s, NCH_2_); 119.39 (s, NCH); 123.90 (s); 125.17 (s); 125.23 (s); 126.54 (s); 126.65 (s); 127.31 (s); 131.29 (s); 131.75 (s); 139.71 (s); 140.23 (s); 140.37 (s);140.44 (s); 140.53 (s); 147.28 (s, NCCH). ^29^Si NMR (CDCl_3_, 59 MHz): −8.05 (Si); 0.38 (Si–CH_3_); 0.42 (Si–CH_3_); 1.54 (Si(CH_3_)_2_).

Elemental analysis. Calcd. (%) for C_880_H_876_N_48_Si_29_: C 81.69, H 6.82, N 5.20, Si 6.29; found: C 81.30, H 6.69, N 5.08, Si 6.11

### 3.3. Characterization

^1^H, ^13^C and ^29^Si NMR spectra were recorded at room temperature on Bruker Avance 300 and Bruker Avance 400 NMR spectrometers using the standard pulse sequences of the Bruker software. Chemical shifts are given in parts per million (ppm). The solvent residual signals (CHCl_3_ (^1^H); CDCl_3_ (^13^C)) were used as internal standards (δ 7.27 (^1^H); δ 77.00 (^13^C)).

The elemental analysis was carried out on a Carlo Erba 1106 with a CHN analyzer.

Thermogravimetric analysis (TGA) was carried out on a Shimadzu-DTG-60H (Japan) at a heating rate of 10°/min in air and argon.

Differential scanning calorimetry (DSC) measurements were performed on a DSC3 (Mettler Toledo, Switzerland) at a heating rate of 20°/min in argon.

GPC analysis was performed on the chromatographic system “Shimadzu” (Japan, Germany) with the detector: refractometer RID-20A; the column: Phenogel 10,000 Å (Size (300 × 7.8 mm)); the standard: polystyrene; the eluent: THF; the temperature: 40 °C; and the speed of flow: 1 mL/s.

The preparative chromatographic system consisted of a high-pressure isocratic pump (SHIMADZU, LC-20AT), a RIDK-102 refractometric detector (Czechia), and 300 × 21.2 mm Phenomenex preparative columns (USA) packed with the Phenogel sorbent (particle size 10 μm). Depending on the molecular masses of the components of the mixtures being separated, columns with pore sizes of 103, 104, and 105 Å were used and THF was used as the eluent.

Mass spectral analysis was carried out using a Shimadzu AXIMA Confidence MALDI-TOF mass spectrometer. To prepare the sample, separate solutions of the dendrimer and the matrix (2,5-dihydroxybenzoic acid) in THF with a concentration of 1 mg/mL and sodium chloride solution in deionized water with a concentration of 1 mg/mL, or a matrix (α-cyano-4-hydroxycinnamic acid) in a mixture of acetonitrile and 0.1% trifluoroacetic acid (1:1) and a Lithium chloride solution in deionized water with a concentration of 1 mg/mL were mixed and applied to a steel target to dry.

## 4. Conclusions

The synthesis of new hybrid dendrimers composed of HPB units as a rigid polyphenylene shell and a flexible carbosilane dendritic core linked through the triazole cycle was accomplished via the “click” reaction of azide-terminated carbosilane cores and ethynyl functionalized HPB units. The effective “click” approach allowed us to obtain the desired dendrimers of high purity and yield. Surprisingly, the dendrimers showed a similar reversible phase behavior regardless the generation number, as well as the ability of macromolecules to crystallize with the formation of a columnar phase after annealing at a temperature above the melting point. We propose similar thermal properties regardless of the generation to the nearly same ratio of rigid and flexible blocks for both G1 and G2 dendrimers. The crystallinity of dendrimers was estimated as 50–57% and, according to DSC, was a slightly higher for G1 than for G2. We assume that this was due to a somewhat higher content of crystallizable units with regard to the amorphous units in case of G1.

According to SWAXS experiments, the HPB blocks in the dendrimer termini promoted the formation of a stable crystal structure with a regular crystalline columnar organization. However, the removing of one benzene group from the HPB unit distorts the system and its propensity to crystalize due to increasing the molecular torsion mobility.

One of the main conclusions drawn from this study is that hybrid dendrimers are prone to ordering depending on their constituents of different nature, while for homogeneous dendrimers, the propensity to order is determined by the dendrimer molecule as a whole. We believe the sophisticated designing of dendrimers paves the way for the development of regular macromolecular structures with predicted molecular characteristics.

## Figures and Tables

**Table 1:** Parameters of the phase behavior of dendrimers in heating–cooling cycles.

Cycle		G1					G2				
		T_cc_, °C	T_melt_, °C	ΔH_melt_, J/g	T_cryst_, °C	ΔH_cryst_, J/g	T_cc_, °C	T_melt_, °C	ΔH_melt_, J/g	T_cryst_, °C	ΔH_cryst_, J/g
1	Heating	213	306	31	-	-	212	312	23	-	-
	Cooling	-	-	-	220/240	29	-	-	-	225	19
2	Heating	-	273	31	-	-	-	276/311	21/23	-	-
	Cooling	-	-	-	216	25	-	-	-	223	19
3	Heating		271	29	-	-		273/303	20/22	-	

## Data Availability

The data presented in this study are available upon request from the corresponding author.

## Supplementary Materials

The following supporting information can be downloaded at: https://www.mdpi.com/article/10.3390/ijms232415461/s1. The supporting information contains NMR spectra, GPC curves, MALDI-ToF mass-spectrum, schemes of synthesis of intermediate carbosilane dendrimers, and HPB monodendron.
