# Supplementary material for: Controllable Synthesis of Hybrid Dendrimers Composed of a Carbosilane Core and an Aromatic Shell: Does Size Matter?
Source: Int J Mol Sci. 2022 Dec 7;23(24):15461. doi: 10.3390/ijms232415461 (PMC9779566; doi:10.3390/ijms232415461)
Supplement: Supplementary file 1 [file ijms-23-15461-s001.zip › ijms-2055536-supplementary.pdf]

# Controllable synthesis of hybrid dendrimers composed of a carbosilane core and an aromatic shell: Does size matter?

Sofia N. Ardabevskaia [1,2], Elena S. Chamkina [3], Irina Yu. Krasnova [3], Sergey A. Milenin [1,2], Ekaterina A. Sukhova [3], Konstantin L. Boldyrev [3], Artem V. Bakirov [1,4], Olga A. Serenko [3], Zinaida B. Shifrina [3,*], Aziz M. Muzafarov [1]

[1] N.S. Enikolopov Institute of Synthetic Polymeric Materials, Russian Academy of Sciences, 70 Profsouznaya St., Moscow,117393, Russia

[2] Tula State Lev Tolstoy Pedagogical University, Research laboratory of new silicone materials and technologies, 125 Lenin Ave., building 4, 300026 Tula, Russia

[3] A.N. Nesmeyanov Institute of Organoelement Compounds, Russian Academy of Sciences, 28 Vavilov St., Moscow, 119991, Russia

[4] National Research Center “Kurchatov Institute”, Akademika Kurchatova pl., 1, 123182 Moscow, Russia

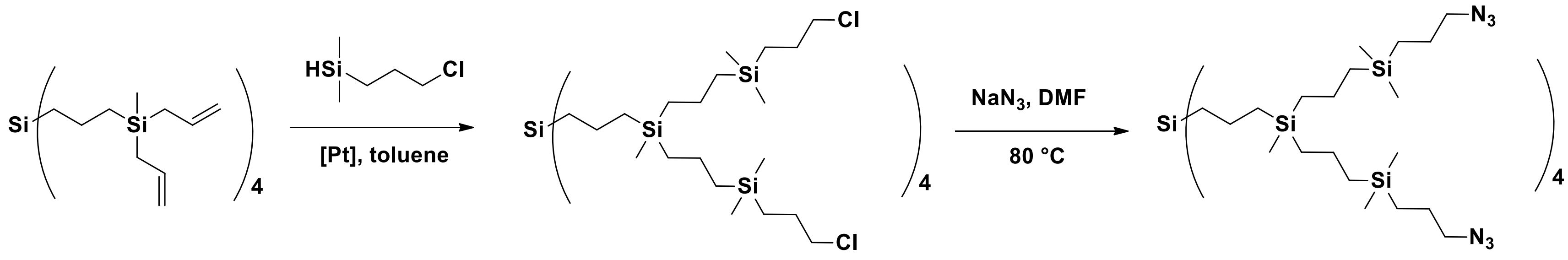


Scheme S1. Synthesis of dendrimers $G1Si_{13}(Cl)_8$ and $G1Si_{13}(N_3)_8$

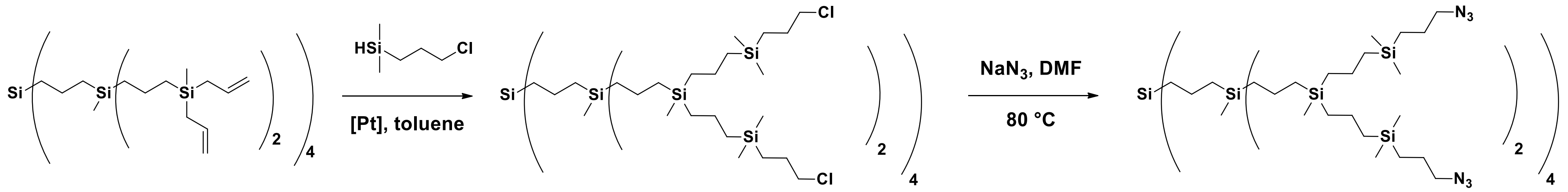


Scheme S2. Synthesis of dendrimers $G2Si_{29}(Cl)_{16}$ and $G2Si_{29}(N_3)_{16}$

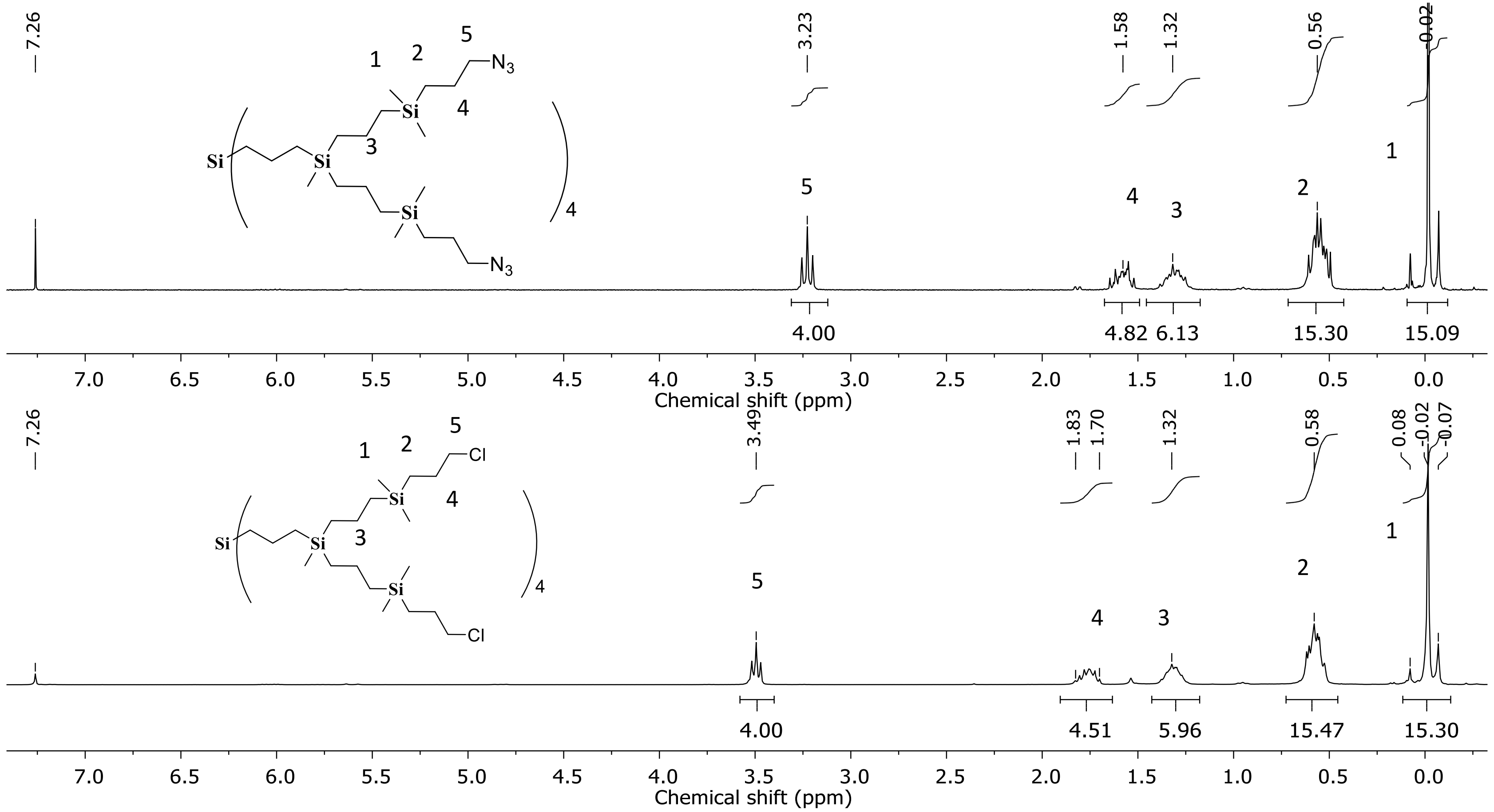


Figure S1. $^{1}$H NMR spectra of dendrimers $G1Si_{13}(Cl)_8$ and $G1Si_{13}(N_3)_8$

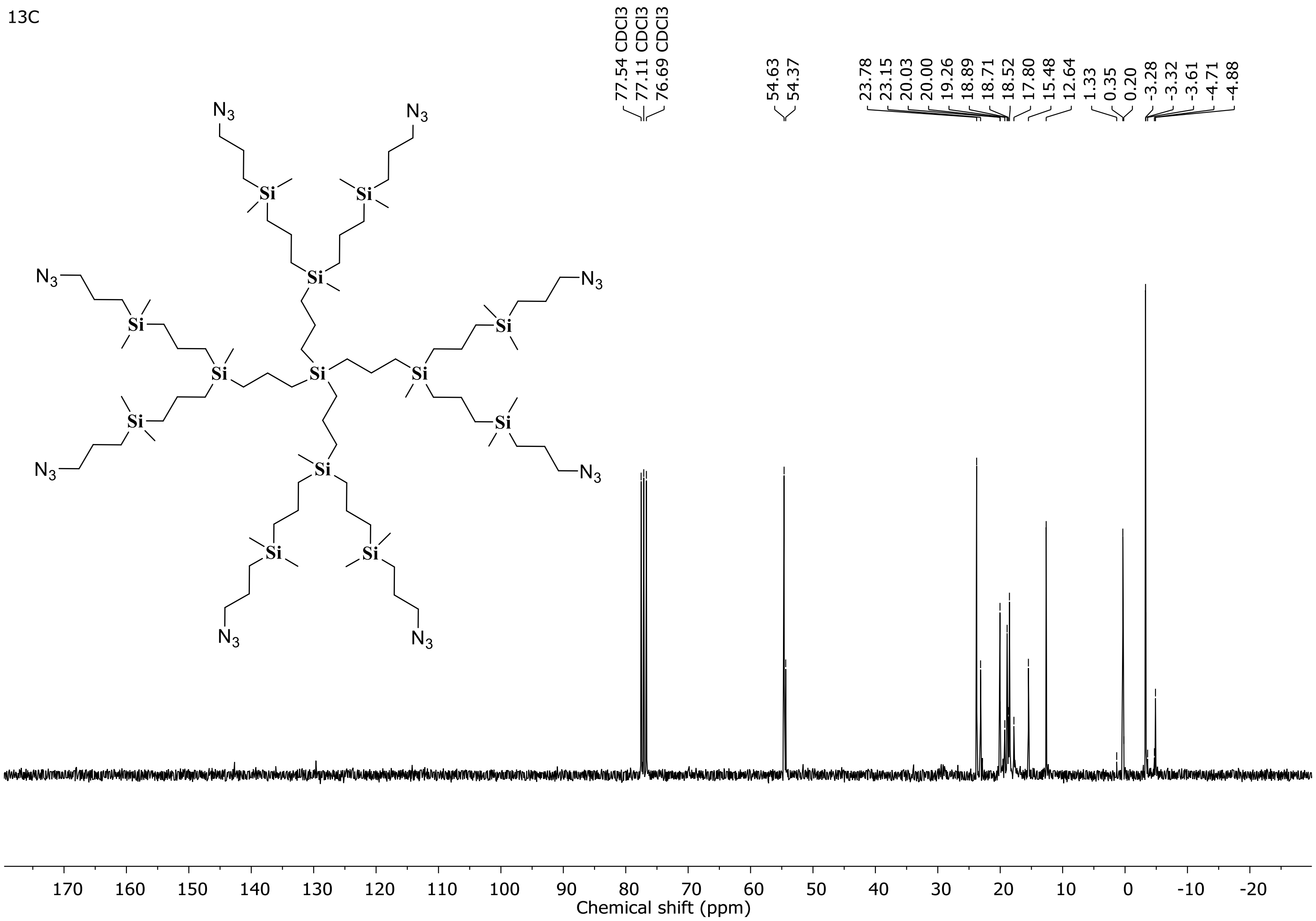


Figure S2. $^{13}$C NMR spectrum of dendrimer $G1Si_{13}(N_3)_8$

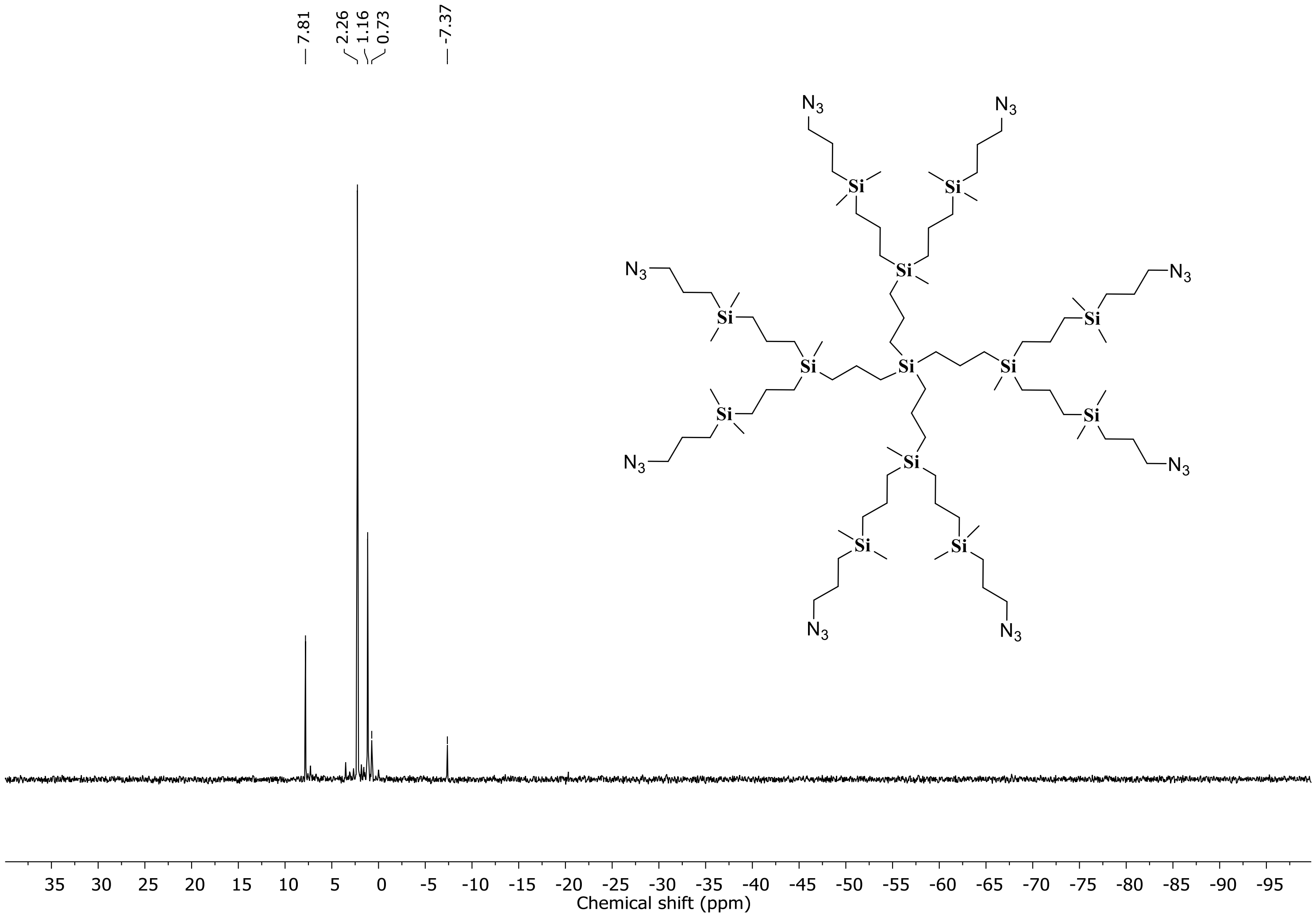


Figure S3. $^{29}$Si NMR spectrum of dendrimer $G1Si_{13}(N_3)_8$

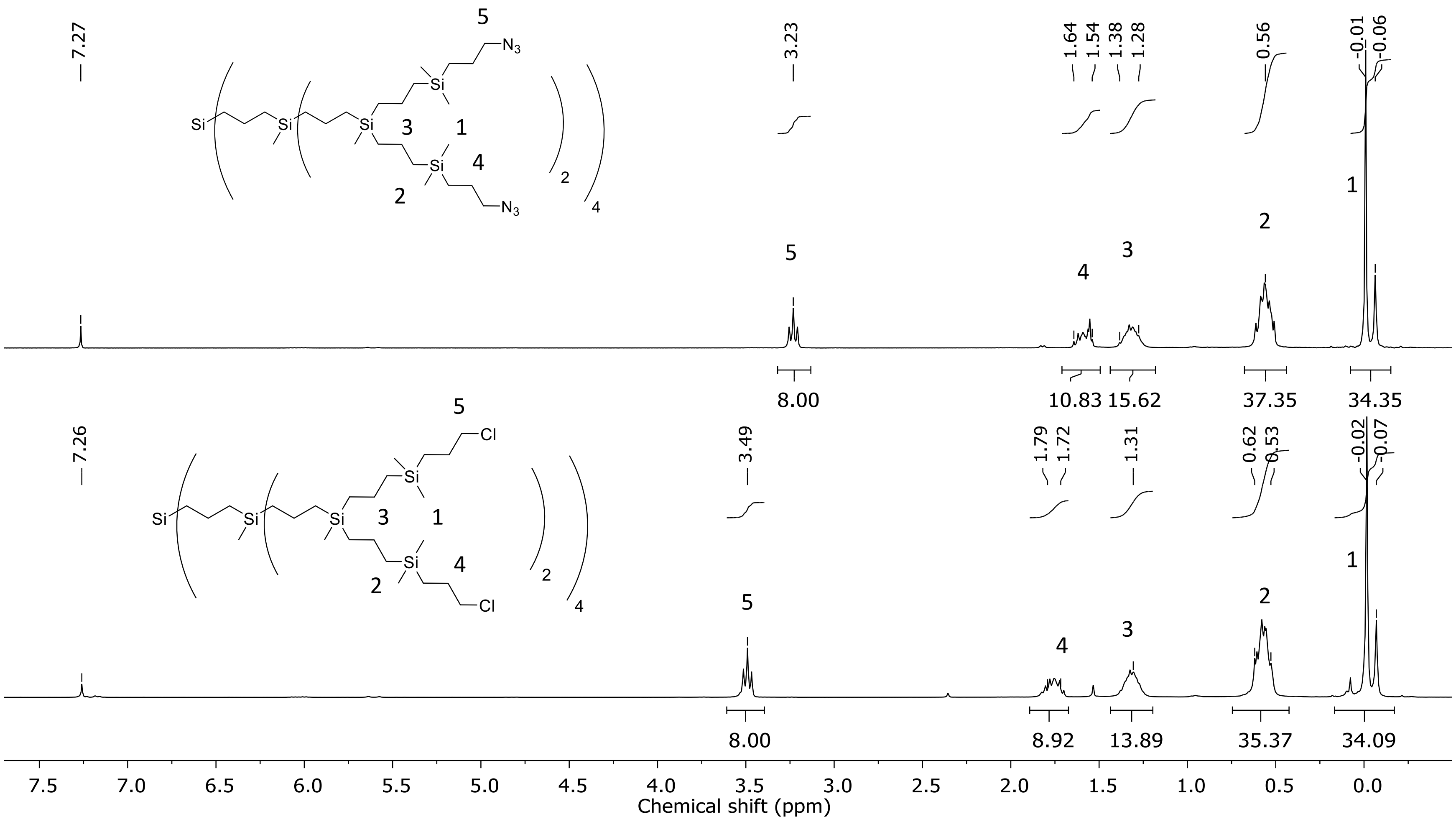


Figure S4. $^{1}H$ NMR spectra of dendrimers G2Si$_{29}$(Cl)$_{16}$ and G2Si$_{29}$(N$_3$)$_{16}$

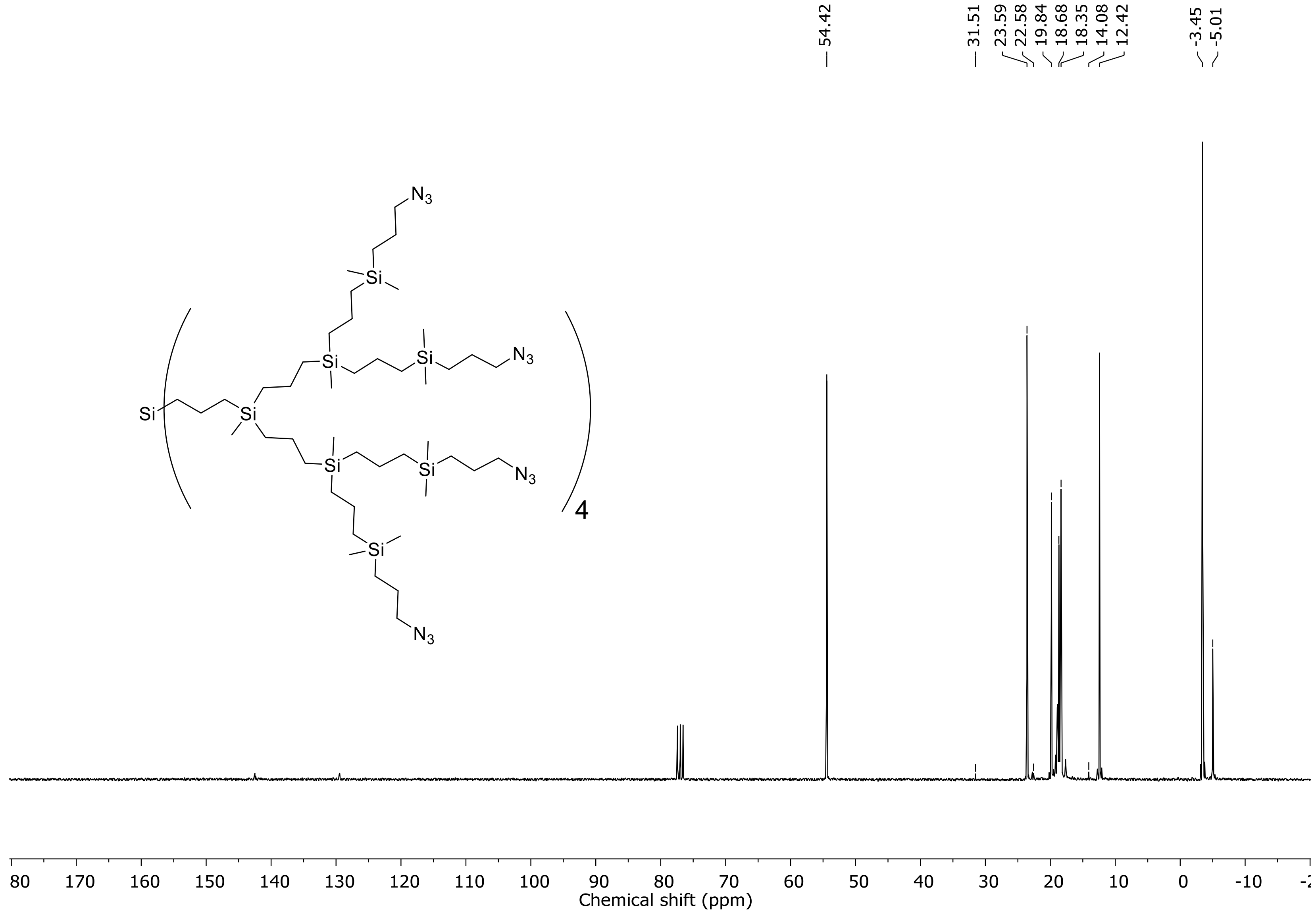


Figure S5. $^{13}$C NMR spectrum of dendrimer $G2Si_{29}(N_3)_{16}$

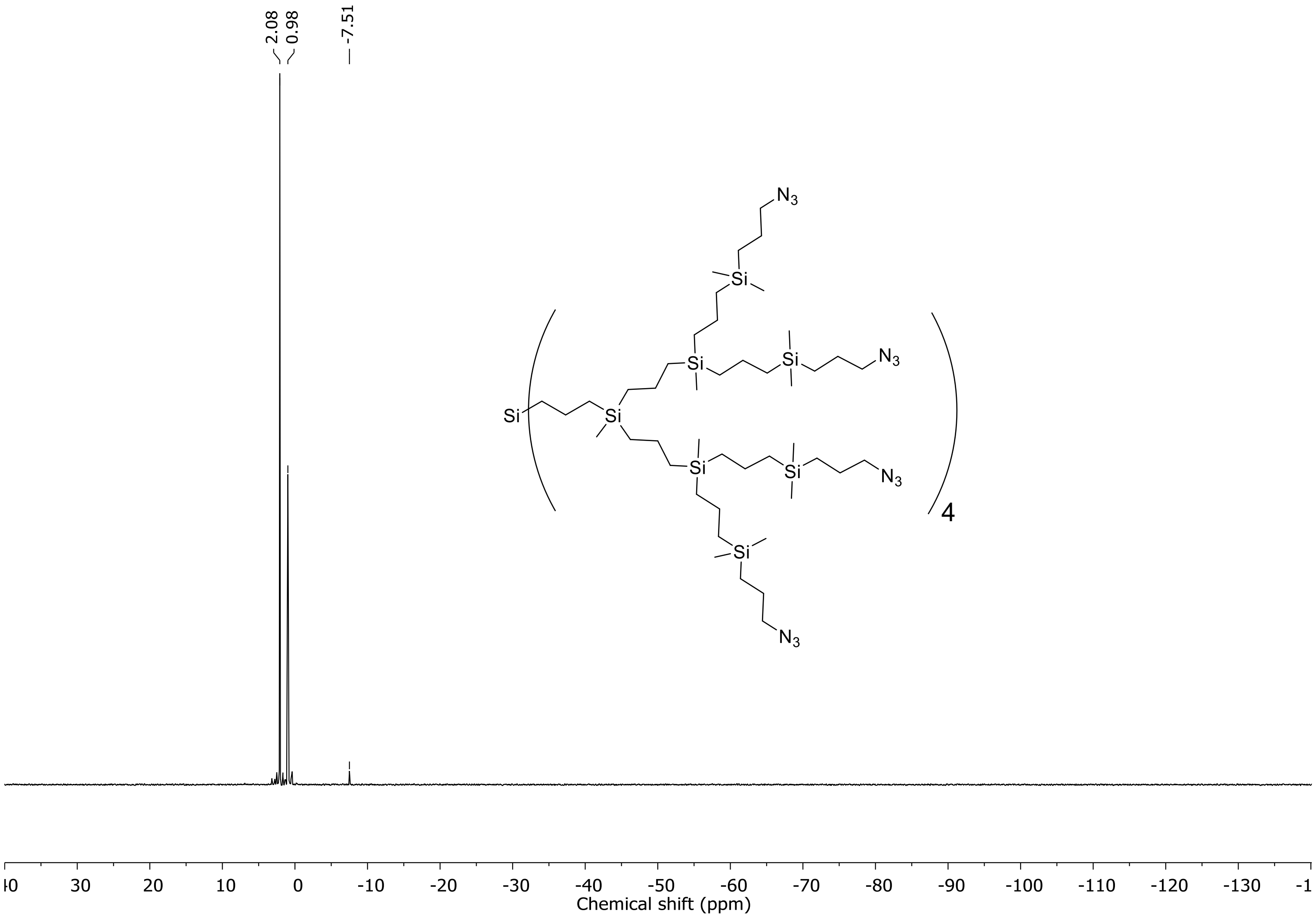


Figure S6. $^{29}$Si NMR spectrum of dendrimer G2Si$_{29}$(N$_3$)$_{16}$

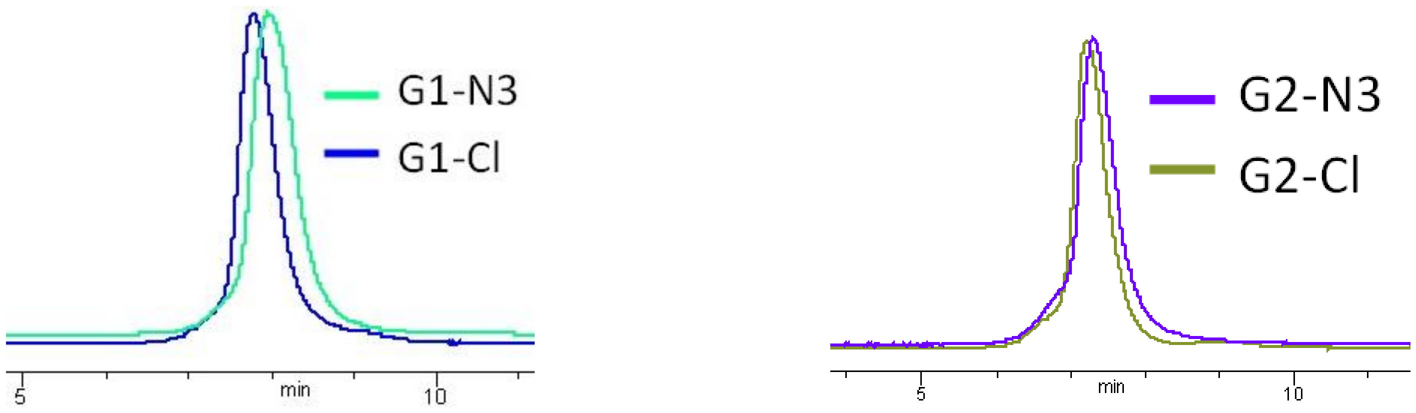


Figure S7. GPC curves of chlorine- and azide-terminated dendrimers

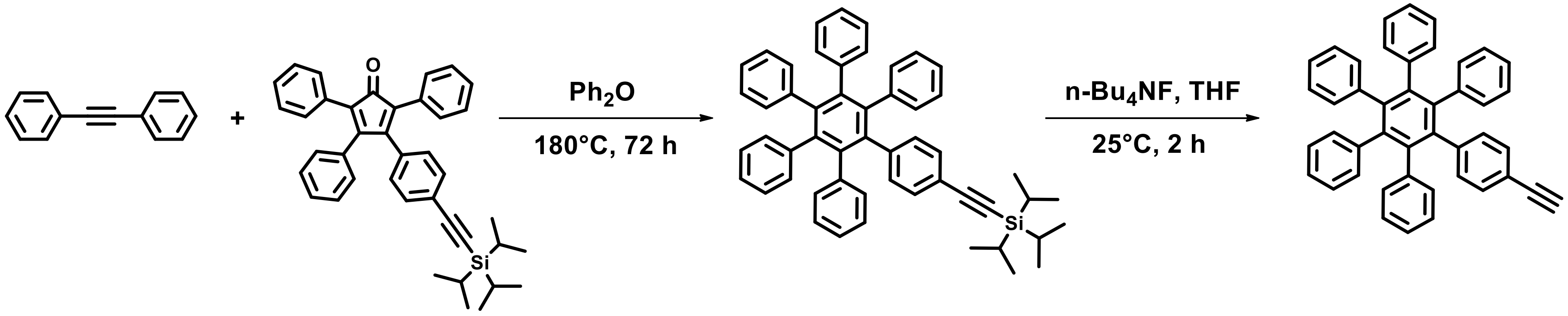


Scheme S3. Synthesis of monoethynyl-containing HPB dendron

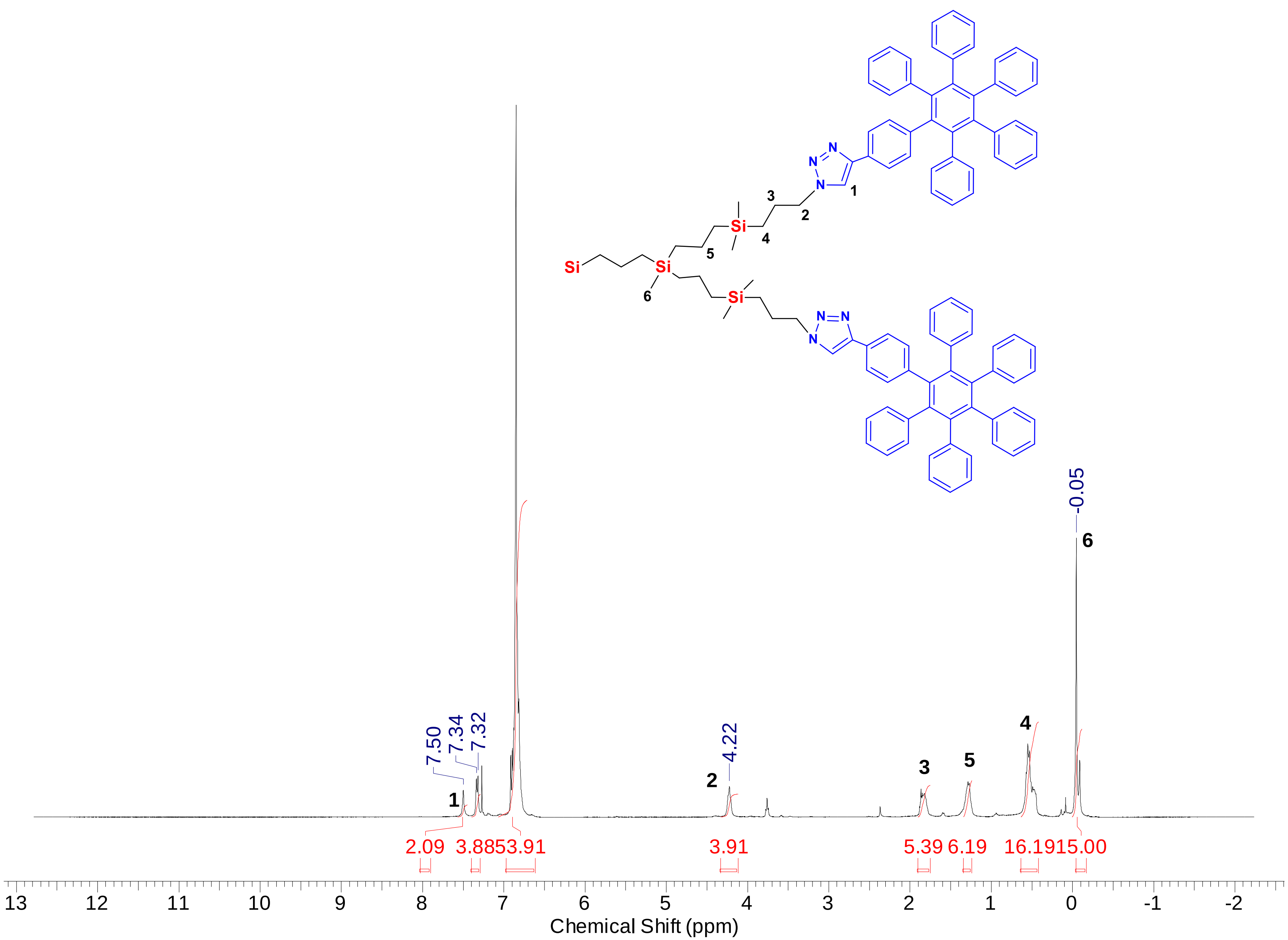


Figure S8. $^1$H NMR spectrum of dendrimer ***G1Si$_{13}$Ar$_{56}$***. The integral are given for one branch

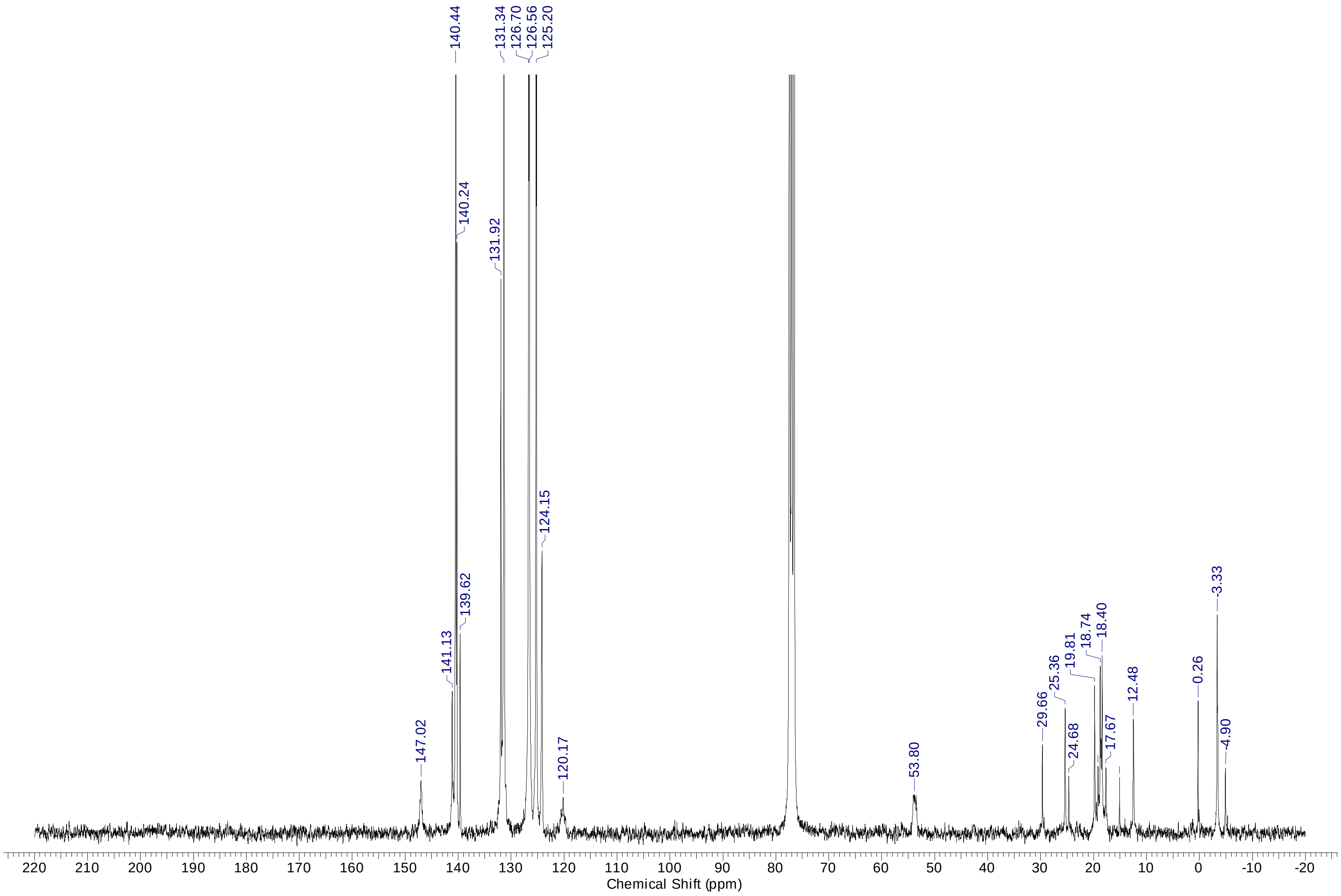


Figure S9. $^{13}$C NMR spectrum of dendrimer ***G1Si$_{13}$Ar$_{56}$***

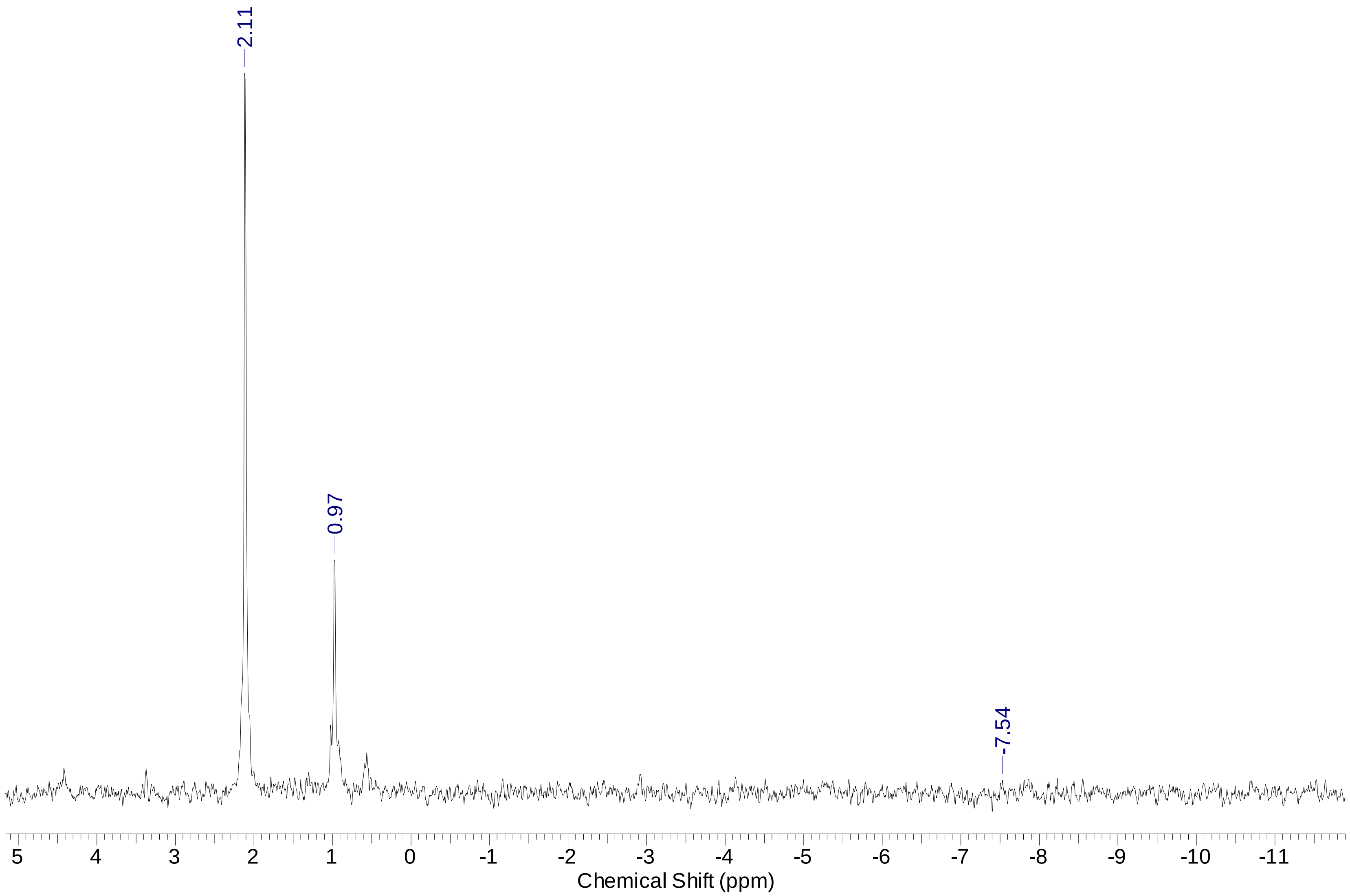


Figure S10. $^{29}$Si NMR spectrum of dendrimer ***G1Si$_{13}$Ar$_{56}$***

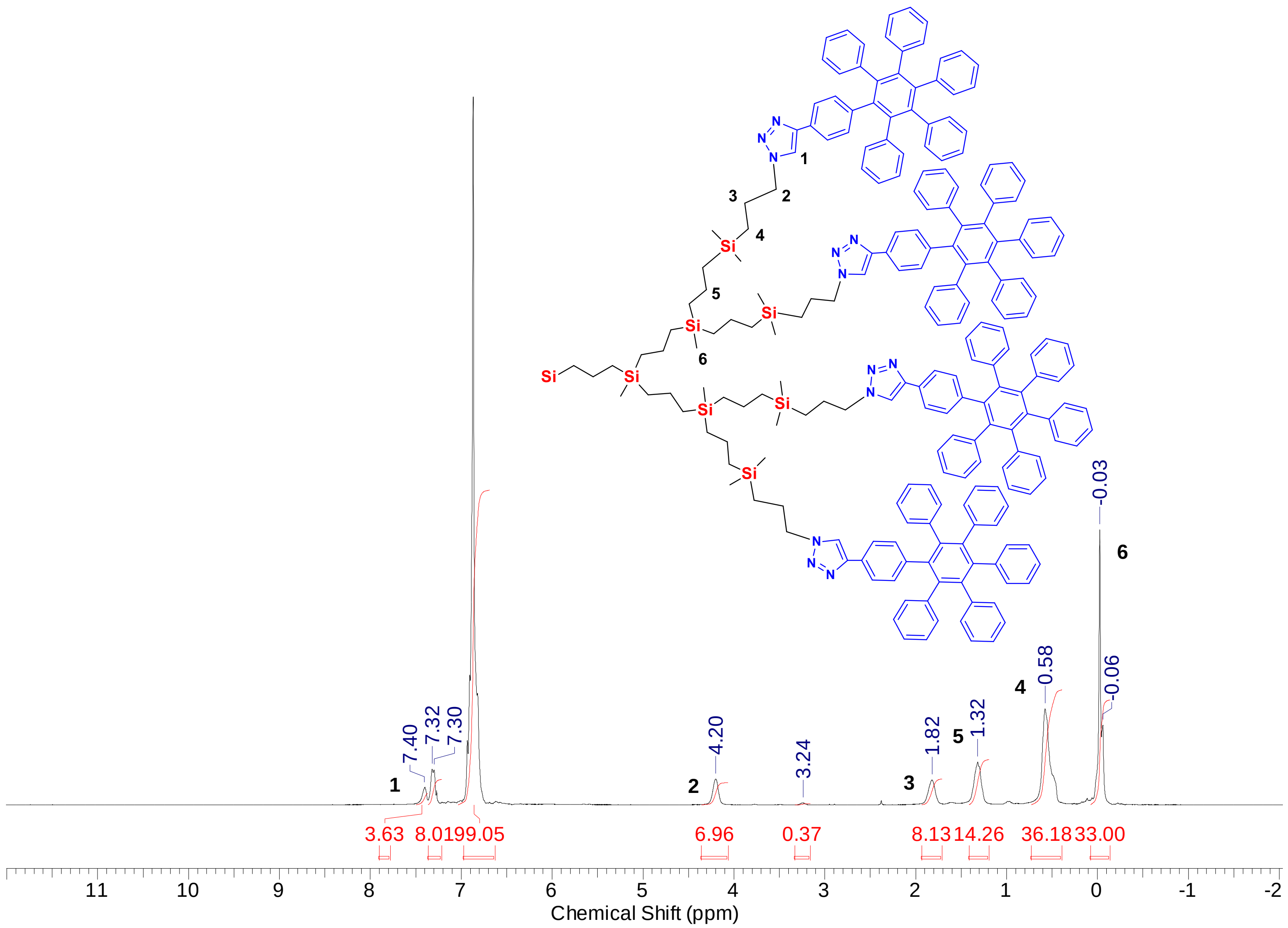


Figure S11. $^{1}$H NMR spectrum of dendrimer ***G2Si$_{29}$Ar$_{112}$***. Reaction conditions: 105°C, 72 h.
The integral values are given for one branch

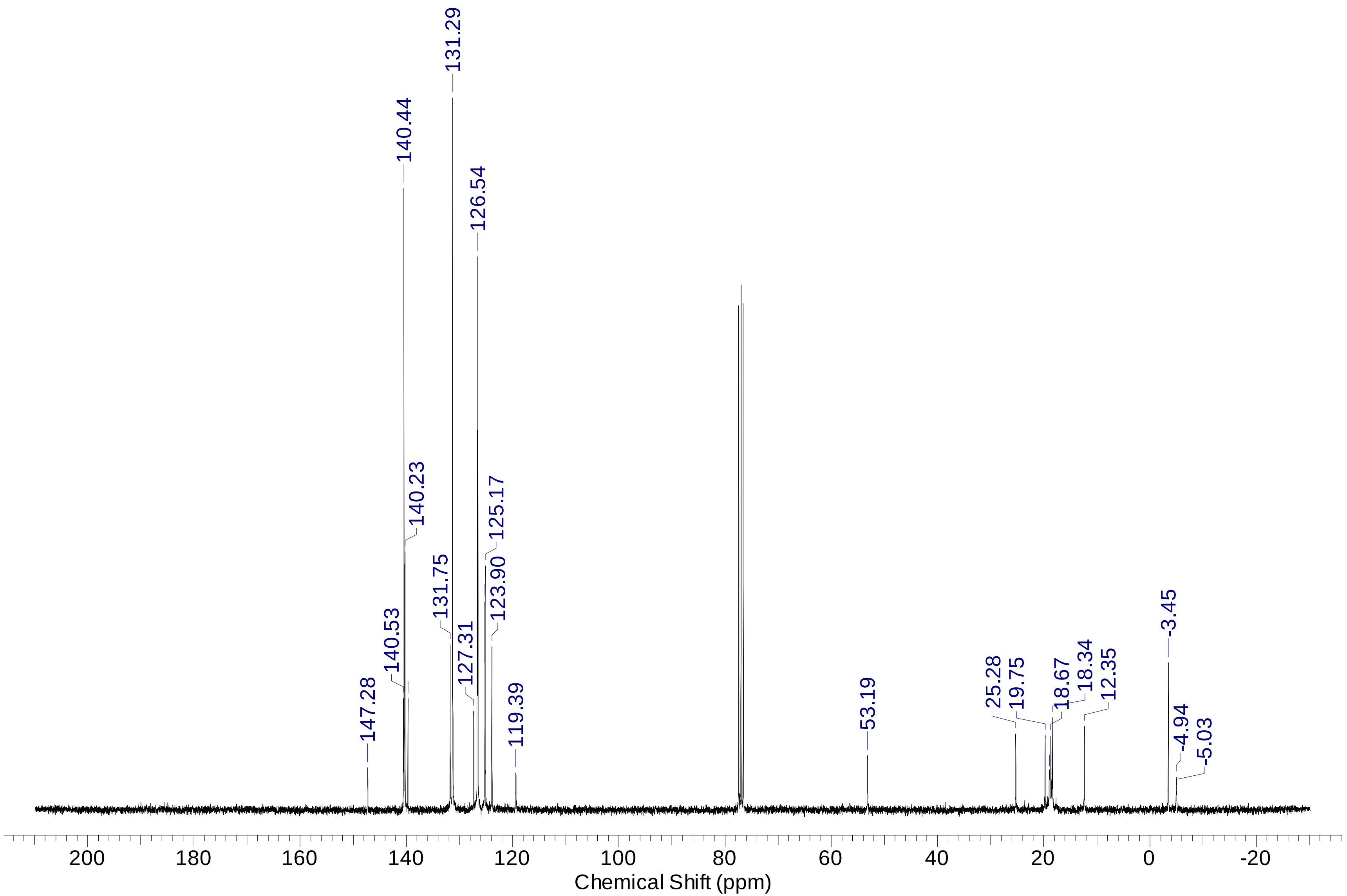


Figure S12. $^{13}$C NMR spectrum of dendrimer ***G2Si$_{29}$Ar$_{112}$***

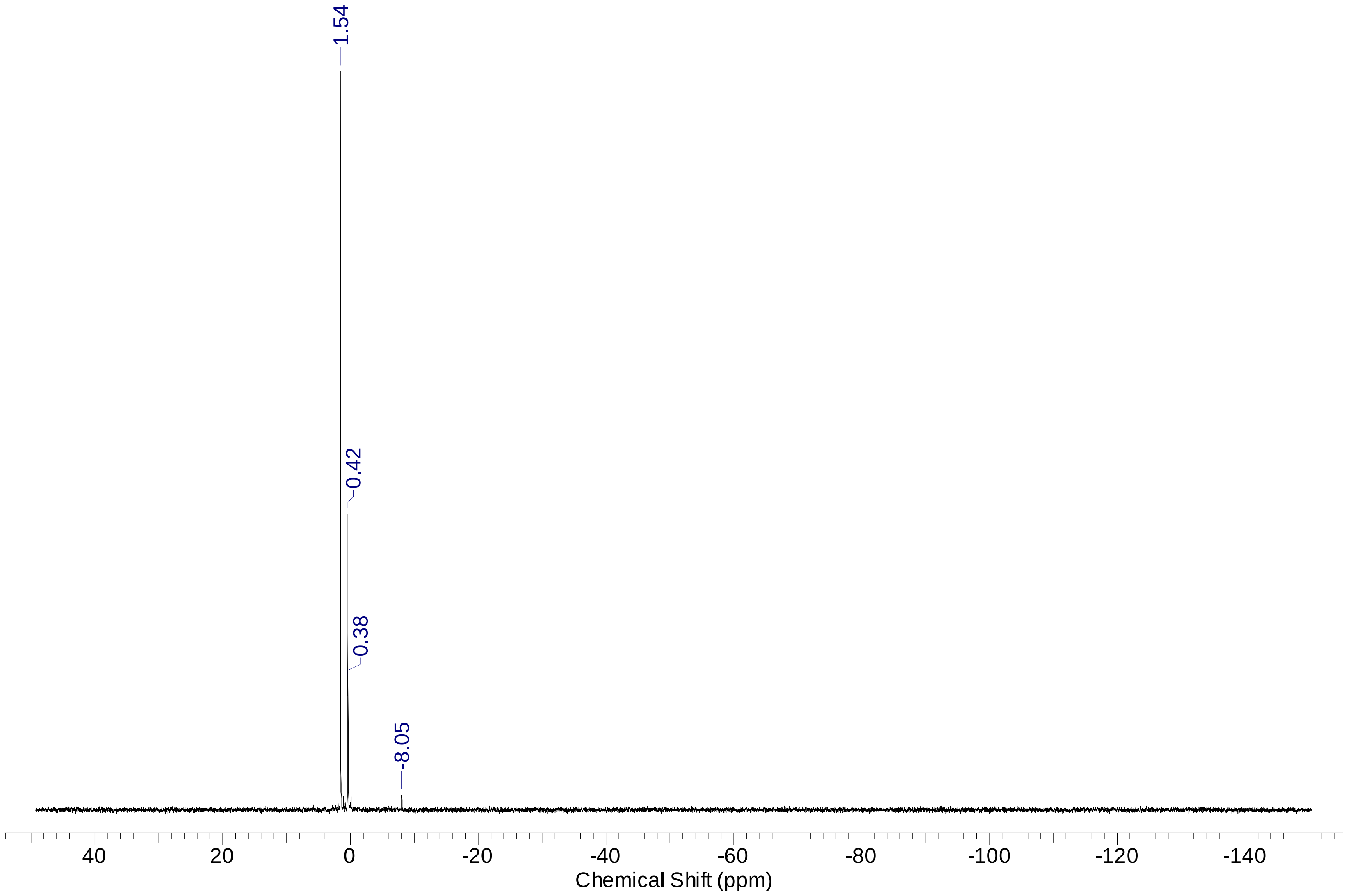


Figure S13. $^{29}$Si NMR spectrum of dendrimer $\boldsymbol{G2Si_{29}Ar_{112}}$

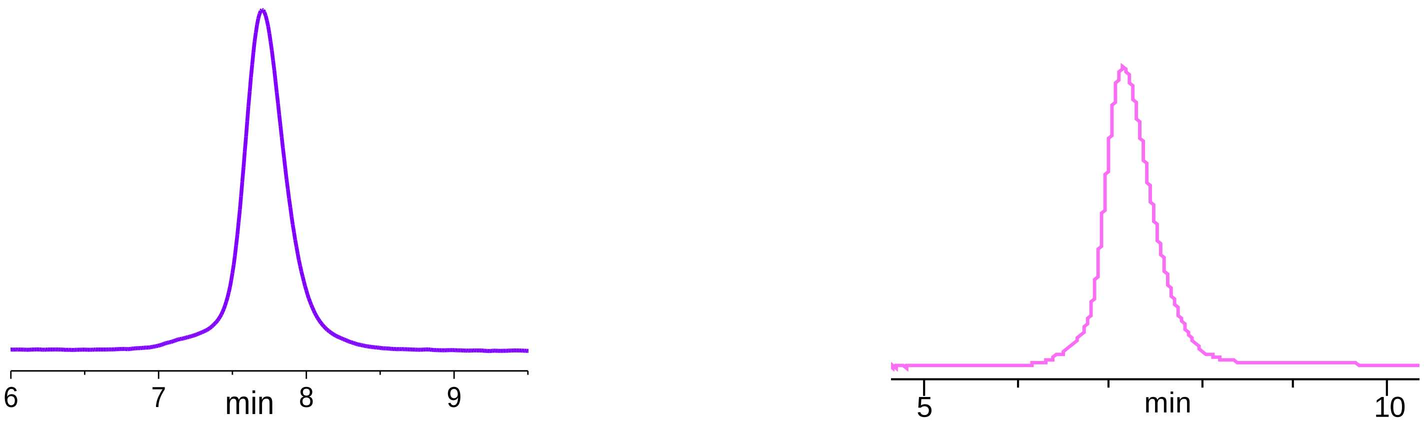


Figure S14. GPC curves of hybrid dendrimers $\boldsymbol{G1Si_{13}Ar_{56}}$ (left) and $\boldsymbol{G2Si_{29}Ar_{112}}$ (right)

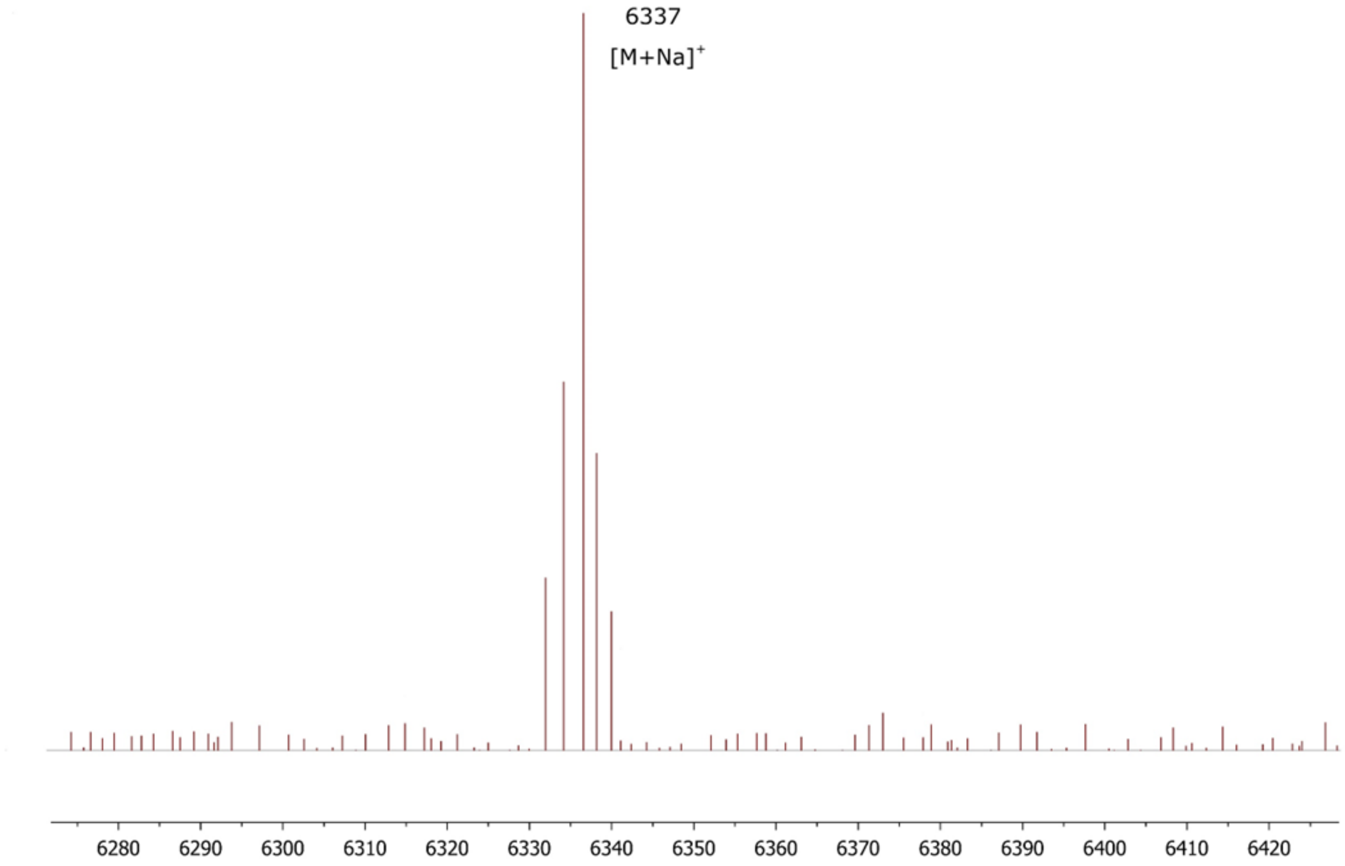


Figure S15. MALDI-ToF mass spectrum of dendrimer ***G1Si$_{13}$Ar$_{56}$***

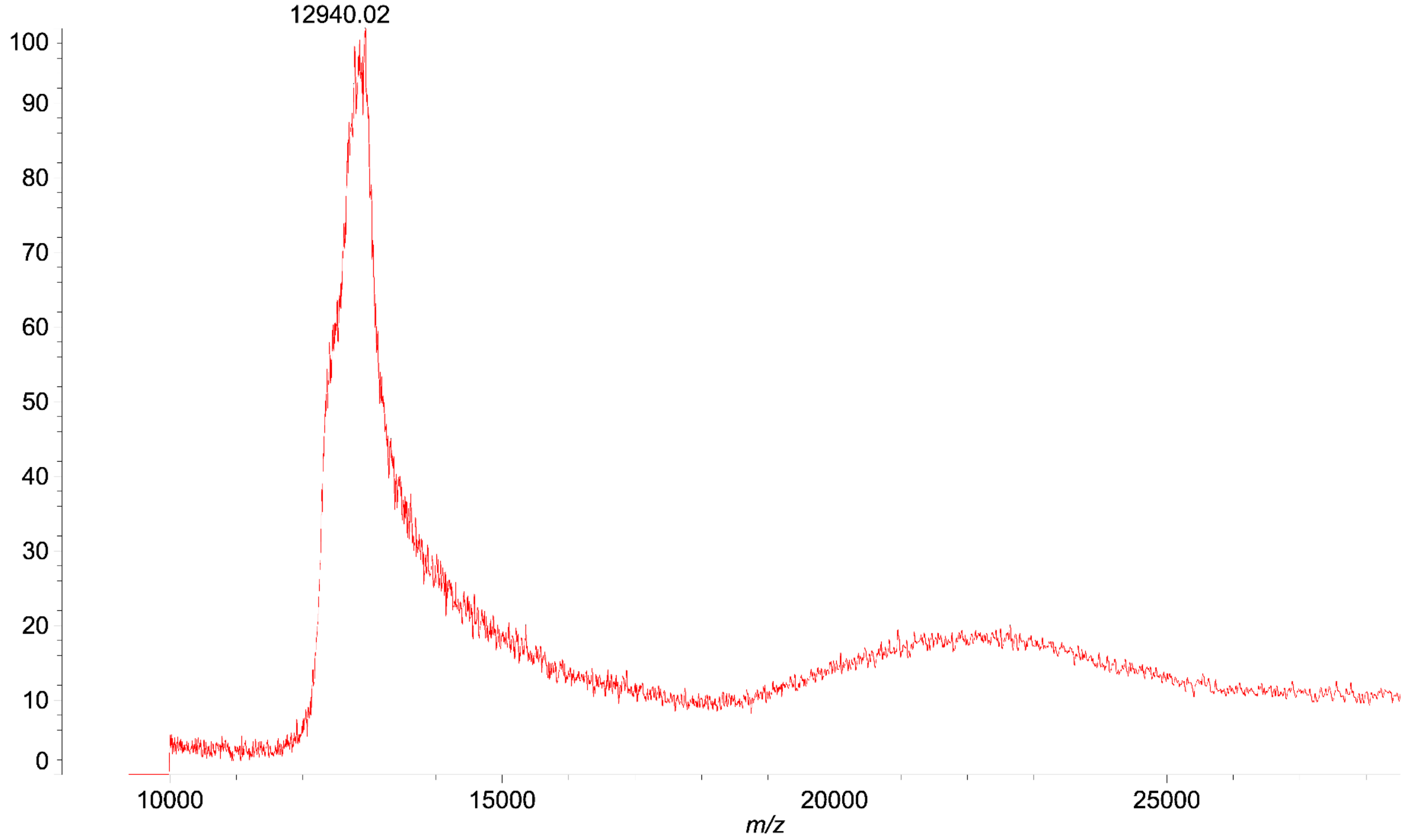


Figure S16. MALDI-ToF mass spectrum of dendrimer ***G2Si$_{29}$Ar$_{112}$***
